# Molecular classification and fertility-sparing outcomes in endometrial cancer and atypical endometrial hyperplasia

**DOI:** 10.17305/bb.2025.12445

**Published:** 2025-08-31

**Authors:** Jiayi Wang, Guozhong Jiang, Shuping Yan, Yanpeng Tian, Yuxi Jin, Hanlin Fu, Lulu Si, Mingbo Cai, Xueyan Liu, Ruixia Guo

**Affiliations:** 1 Department of Obstetrics and Gynecology, The First Affiliated Hospital of Zhengzhou University, Zhengzhou, China; 2 Medical Key Laboratory for Prevention and Treatment of Malignant Gynecological Tumor, Henan, China; 3 Department of Pathology, The First Affiliated Hospital of Zhengzhou University, Zhengzhou, China

**Keywords:** Endometrial cancer, EC, atypical endometrial hyperplasia, AEH, fertility-preserving treatment, molecular classification, fertility outcomes

## Abstract

Molecular classification has emerged as a critical tool for guiding personalized treatment in endometrial cancer (EC) and atypical endometrial hyperplasia (AEH). This retrospective study aimed to assess the impact of molecular classification on fertility-sparing treatment outcomes in patients diagnosed with EC and AEH who underwent fertility preservation therapy between 2006 and 2021. Patients were categorized into four molecular subtypes using immunohistochemistry (IHC) and Sanger sequencing, based on the Proactive Molecular Risk Classifier for Endometrial Cancer (ProMisE): *POLE*-ultramutated, mismatch repair (MMR) deficient (MMRd), p53 abnormal (p53abn), and p53 wild-type (p53wt). All patients were evaluated for oncological prognosis and fertility outcomes, with a total of 103 patients included in the analysis. Recurrence rates exhibited significant differences among the molecular classifications, with the lowest recurrence rate observed in the p53wt subtype (19.7%), followed by MMRd (30.4%), *POLE*-ultramutated (66.7%), and p53abn (71.4%) subtypes. Multivariate Cox regression analysis indicated that the p53abn subtype was a significant risk factor for recurrence following conservation therapy when compared to the p53wt subtype. Additionally, there was a notable disparity in standard surgical treatment due to treatment failure, with operation rates of 7.5%, 19.2%, 66.7%, and 57.1% for the p53wt, MMRd, *POLE*-ultramutated, and p53abn subtypes, respectively. Regarding fertility outcomes, the p53wt group demonstrated the highest pregnancy rate after achieving a complete response compared to the other subtypes; however, no significant differences were observed in overall pregnancy outcomes. The ProMisE molecular classification holds significant prognostic value for patients with EC and AEH undergoing fertility-sparing treatment. Among the molecular subtypes, p53wt appears to be the most favorable for fertility-preserving interventions. This study provides essential insights into reproductive outcomes for this patient population.

## Introduction

Endometrial cancer (EC) is a gynecological malignancy prevalent worldwide. It is the most common malignant tumor of the female reproductive system in developed countries and the second most common in China [1–3]. Although the majority of patients are postmenopausal, up to 14% are women of childbearing age [4]. Atypical endometrial hyperplasia (AEH) is a precancerous endometrial lesion. Approximately 30% of women with complex AEH progress to EC, and up to 40% of women diagnosed with complex atypical hyperplasia develop occult EC during hysterectomy [5, 6]. According to the 2018 National Comprehensive Cancer Network (NCCN) guidelines, the standard treatment for early-stage EC involves total hysterectomy and bilateral salpingo-oophorectomy, with or without dissection of the pelvic and para-aortic lymph nodes [7]. However, these surgical procedures inevitably lead to permanent infertility. Nonetheless, there is a growing desire among patients with EC/AEH to preserve their reproductive function. Fertility-preserving treatments for EC/AEH have shown favorable clinical efficacy, with complete response (CR) rates ranging from 60% to 98% after high-dose progesterone therapy. However, the recurrence rates remain high, varying from 20% to 50% [8–10]. Successful pregnancy is also an important indicator of successful treatment, with previous studies reporting pregnancy rates ranging from 28.8% to 52% [11–13]. However, effective predictors of tumor and fertility outcomes in patients undergoing fertility-preserving treatments are lacking.

In 2013, The Cancer Genome Atlas (TCGA) proposed a molecular classification system for EC based on multi-group sequencing data. This classification system divides EC into four subtypes: *POLE*-ultramutated, microsatellite instability-high (MSI-H), copy number-low (CNL), and copy number-high (CNH). Among these subtypes, patients with the *POLE*-ultramutated subtype exhibited the best prognosis, whereas those with the CNH subtype had the worst prognosis [14]. In 2015, Talhouk et al. simplified this classification by introducing the Proactive Molecular Risk Classifier for Endometrial Cancer (ProMisE) classification system. ProMisE is a clinically relevant and relatively inexpensive subtype classification system based on mismatch repair (MMR) protein, p53 protein, and *POLE* gene testing. It categorizes EC into *POLE*-ultramutated, MMR deficient (MMRd), p53 abnormal (p53abn), and p53 wild-type (p53wt) subtypes [15]. This molecular classification was incorporated into the World Health Organization (WHO) classification of female genital tumours (5th edition), European Society of Gynecologic Oncology-European Society of Radiological Oncology-European Society of Pathology, and NCCN guidelines [16–18]. Consequently, the use of molecular classification in EC research has gained significant attention and is now considered the standard therapy for patients. However, studies on the application of molecular classification in patients with EC/AEH undergoing fertility preservation therapy are limited. Additionally, previous studies on patients receiving fertility-preserving treatments have primarily focused on prognostic outcomes without considering fertility outcomes [19–23]. Therefore, we incorporated molecular classification into this study to determine its effect on prognostic and fertility outcomes in patients with EC/AEH receiving fertility-sparing treatment, providing guidance for future clinical decision-making.

## Materials and methods

### Case selection

Data from patients diagnosed with endometrial endometrioid carcinoma (EEC)/AEH at the First Affiliated Hospital of Zhengzhou University between 2006 and 2021 were retrospectively collected. The diagnosis of EC/AEH was confirmed through histopathological examination following dilation and curettage with hysteroscopy. The staging of EC followed the FIGO staging guidelines [7], and the grading was evaluated according to the 2014 WHO criteria [24]. All pathological examinations were centrally reviewed by the Pathology Department of the First Affiliated Hospital of Zhengzhou University. Diagnoses were independently reviewed by two gynecological oncology pathology experts with associate senior professional titles or higher, each with over 15 years of professional experience. In cases of differing opinions, a consensus was reached through discussion within the pathology department to establish a final diagnosis. This study was approved by the Ethics Committee of the First Affiliated Hospital of Zhengzhou University (2023-KY-0483-001).

The inclusion criteria for this study, based on the NCCN guidelines, were as follows: (1) confirmation of AEH or well-differentiated (grade 1) EC; (2) localized disease confined to the endometrium, validated by imaging, preferably magnetic resonance imaging (MRI), although expert transvaginal ultrasound (TVS) was deemed acceptable; (3) absence of suspicious lesions or metastases in the images; (4) participants aged between 18 and 40 years; and (5) absence of contraindications due to medication or pregnancy. All patients were informed that fertility-preserving treatments were not the standard approach for EC. Patients with poor tumor tissue quality or insufficient samples were excluded from the study.

General patient information was collected, including age, height, weight, reproductive history, comorbidities, family history, and blood pressure. Prior to treatment, blood samples were collected from patients and analyzed for glucose, insulin, lipid, and serum CA125 levels. The analysis of serum samples was conducted in the laboratory of the Department of Laboratory Medicine at the First Affiliated Hospital of Zhengzhou University.

Body mass index (BMI) was calculated by dividing the weight (kg) by the square of height (m^2^). Metabolic syndrome was defined based on previous studies [25]. Polycystic ovary syndrome (PCOS) was diagnosed based on the Rotterdam consensus criteria [26].

### Hormonal therapy

Patients who met the inclusion criteria received counseling regarding the potential side effects of hormone therapy and the risk of disease progression and relapse. Following the NCCN guidelines, a personalized treatment plan was developed for each patient, which typically included oral administration of high-potency progesterone (megestrol acetate/medroxyprogesterone acetate [MA/MPA]) and the insertion of an intrauterine device with levonorgestrel (LNG-IUS) [7]. In cases where progestins were contraindicated, patients might consider using GnRH-a in combination with aromatase inhibitors or traditional Chinese medicine adjuvant therapy. For patients with PCOS or obesity, combination therapy with metformin was used to improve insulin resistance, given metformin’s status as the preferred antidiabetic agent for patients with type 2 diabetes. During treatment, each patient underwent a comprehensive weight-loss regimen that included dietary modifications, exercise recommendations, as well as careful monitoring of medication compliance and potential side effects.

### Efficacy evaluation

The efficacy evaluation of AEH/EEC was based on established criteria from previous studies [27, 28]. These criteria included the following classifications: (1) CR, which indicated the absence of AEH or EEC lesions in the histopathological analysis; (2) partial response (PR), characterized by a reduction in the crowding of endometrial glands, with the possibility of residual structures such as papillae and cribriform patterns, alongside a decrease in the abnormality of adenoepithelium; (3) stable disease (SD), indicating no change in tumor tissue compared to pretreatment pathological results; (4) progressive disease (PD), characterized by an increase in histopathological grade of tumors and cellular heterogeneity; and (5) recurrence, indicating the reappearance of pretreatment lesions (AEH or EEC) in specimens after achieving CR. Time to CR was calculated from the initiation of hormonal treatment, time to recurrence was calculated from the date of CR, and time to recurrence was calculated from the date of the first positive biopsy. The follow-up period was defined as the time from the initiation of progesterone treatment to the last recorded clinical visit.

Follow-ups were conducted every 4–6 weeks to monitor disease progression. TVS was used to observe the thickness of the endometrial lining and check for signs of muscular invasion. Pelvic MRI was conducted as necessary to rule out muscle infiltration or metastasis to the lymphatic or ovarian regions. Endometrial tissue samples were collected every 3 months using a hysteroscope to assess the response of the lesions to treatment. After achieving CR, the patients were followed up every 3–6 months.

According to the NCCN guidelines, it is recommended that patients who do not achieve CR after 6–12 months of therapy should receive standard treatment, including hysterectomy [7]. However, for patients who declined hysterectomy, an alternative treatment option was offered based on the recommendations of a multidisciplinary team. Medical history, imaging, and pathological data of all patients were recorded. Once CR was achieved, patients who desired pregnancy were encouraged to conceive or were referred for assisted reproductive technology (ART) when necessary. Patients who achieved CR but did not have a recent pregnancy plan were advised to undergo maintenance therapy to prevent recurrence.

### Molecular classification in endometrial specimens

Endometrial tissue samples were assessed using the ProMisE algorithm and categorized into four subtypes: *POLE*-ultramutated, MMRd, p53abn, and p53wt. Furthermore, the multiple classifiers EC/AEH can be assigned to an appropriate molecular subgroup. As per previous studies by León-Castillo et al. [29, 30], tumors were classified as the *POLE*-ultramutated subtype if they exhibited *POLE* mutations with aberrant expression of the p53 protein and/or MMR protein deletion.

### Immunohistochemical expression of MMR proteins and p53 protein

MMR proteins, including MLH1, MSH2, MSH6, and PMS2, were detected using immunohistochemistry (IHC) according to the two-step EnVision method.

Formalin-fixed paraffin-embedded (FFPE) tissues were sliced, dewaxed, hydrated, antigen-repaired, and blocked with endogenous peroxidase. Subsequently, MLH1 (1:250; ProteinTech Group), MSH2 (1:100; ProteinTech Group), MSH6 (1:100; ProteinTech Group), and PMS2 (1:150; ProteinTech Group) antibodies were applied overnight at 4 ^∘^C. The sections were then washed with PBS and incubated with a biotinylated goat anti-rabbit immunoglobulin antibody. Finally, the samples were observed microscopically after DAB color development, redyeing, dehydration with alcohol, and sealing. Diagnoses were made by two pathologists for all biopsies. We identified MMR proteins in the nucleus and used normal tissues as an internal control.

A negative result was inferred if the tumor nucleus remained unstained while the normal tissue nucleus was stained. Conversely, staining of nuclei from both tumor and normal tissue cells was deemed a positive outcome.

**Figure 1.**
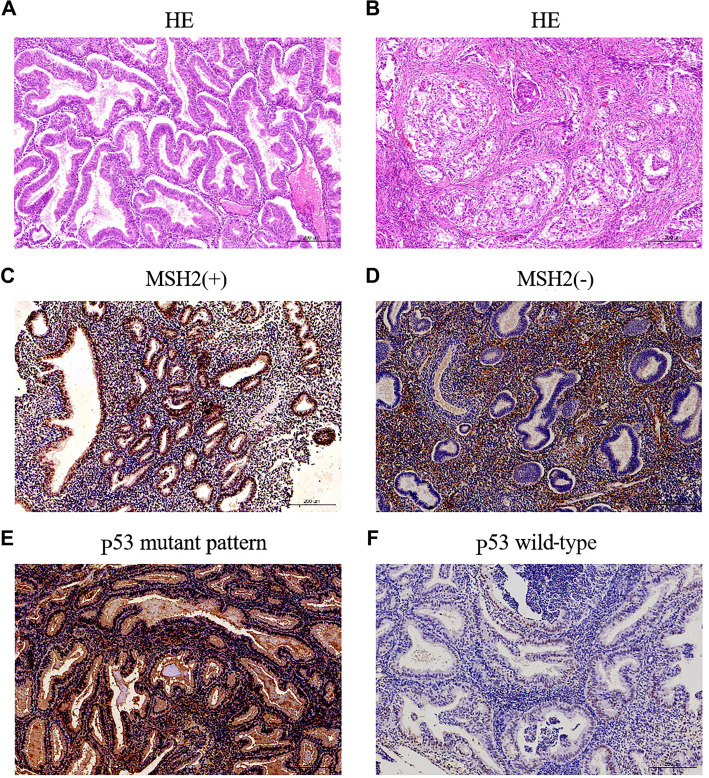
**Representative H&E or IHC images of EEC/AEH.** Representative H&E staining of AEH (A) and EEC (B). MSH2 positive staining (C), MSH2 loss (D), p53 abnormal (p53abn) (E), p53 wild-type (F). Abbreviations: AEH: Atypical endometrial hyperplasia; EEC: Endometrioid endometrial cancer; HE: hematoxylin and eosin.

p53 protein assays were performed using the same IHC method with a p53 primary antibody (1:250; ProteinTech Group). Interpretation of the results considered immunostaining for the p53 protein as aberrant if the tumor nuclei were unstained or showed grossly diffuse patterns (indicating a lack of p53 protein or abnormal protein aggregates, respectively), while intermediate levels of expression were considered wild-type. Representative hematoxylin and eosin (H&E) and IHC images of EECs/AEHs are shown in Figure 1.

**Figure 2.**
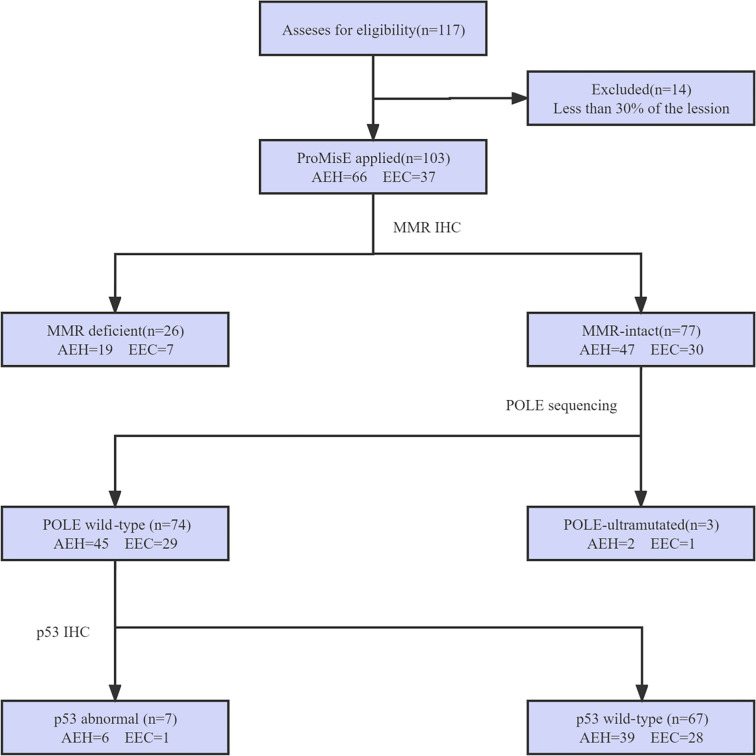
**Flowchart of patient selection and classification using the ProMisE algorithm.** Abbreviations: AEH: Atypical endometrial hyperplasia; EEC: Endometrioid endometrial cancer; MMR: Mismatch repair; IHC: Immunohistochemistry.

### *POLE* mutations detected through Sanger sequencing

PCR amplification and Sanger sequencing were used to evaluate mutations in *POLE* exons 9, 13, and 14. Genomic DNA was extracted from FFPE tissues and amplified using positive and negative primers. PCR products were then purified, dried, and diluted. Sanger sequencing was performed on the purified PCR products using the BigDye Terminator v3.1 sequencing kit (Applied Biosystems) and the automated sequencing instrument 3500Dx (Applied Biosystems). The resulting data were exported and analyzed using Chromas software to compare the sequencing results with the reference sequences. This analysis aimed to identify gene mutations present in the tested segments.

### Ethics approval and consent to participate

The patients provided written informed consent to participate. Written informed consent was obtained from the patients for the publication of the data and images in this case report.

### Statistical analysis

Continuous variables with normal distribution were presented as the mean ± standard deviation (x̅ ± s), and comparisons were made using Student’s *t*-test or one-way ANOVA. Normality was assessed using the Shapiro–Wilk normality test. For non-normally distributed variables, the median and interquartile range (IQR) were calculated, and comparisons were performed using the Mann–Whitney *U* test. Categorical variables were described as rates or percentages (%) and compared using the chi-square test or Fisher’s exact test. Multivariate analysis was performed using the Cox proportional hazards regression test. All statistical analyses were performed using SPSS 25.0 software and R software (version 4.3.2). A statistically significant difference was considered at *P* < 0.05.

## Results

### Comparison of baseline and clinical characteristics among the four molecular subtypes

A total of 117 patients with EEC/AEH who received fertility-sparing treatment at the First Affiliated Hospital of Zhengzhou University between 2006 and 2021 were retrospectively investigated (Figure 2). Out of the initial sample, 14 cases with poor tumor tissue quality or insufficient samples were excluded. Ultimately, 103 patients who met all of the inclusion and exclusion criteria were retrospectively analyzed in this study, including 37 (35.9%) patients with EEC and 66 (64.1%) patients with AEH. The mean age at the time of initial diagnosis was 31.68 ± 4.54 years; the mean BMI was 27.42 ± 3.95 kg/m^2^. Seventy-five (72.8%) patients had never delivered before. The clinical follow-up period ranged from 12 to 201 months, with a median follow-up of 53 months. Fifteen (14.6%) patients were diagnosed with PCOS. Additionally, there were 28 patients (27.2%) with MetS. Regarding treatment regimen, 62 (60.2%) patients received oral MA or MPA, while 38 (36.9%) patients were treated with a combination of MA/MPA and LNG-IUS. Furthermore, 3 patients (2.9%) chose alternative options based on personal preference and medical necessity (Table 1). There was no statistical difference between the four groups in terms of age, BMI, metabolic syndrome, comorbidity with PCOS, fertility history, diagnosis, family history, serum CA125, and choice of treatment regimen (*P* > 0.05).

**Table 1:** Baseline and clinical characteristics across molecular classification

**Variables**	**Total** ***n* (%)**	***POLE-*ultramutated** ***n* (%)**	**MMR deficient** ***n* (%)**	**p53 abnormal** ***n* (%)**	**p53 wild-type** ***n* (%)**	***P* value**
No. of patients	103	3	26	7	67	–
Age (years)	31.68±4.54	35.33±1.53	32.25±4.98	29.57±3.60	31.48±4.46	0.254
BMI (kg/m^2^)	27.42±3.95	26.34±0.88	26.40±4.21	29.51±2.66	27.64±3.97	0.247
Metabolic syndrome						0.701
No	75 (72.8%)	2 (66.7%)	20 (76.9%)	4 (57.1%)	49 (73.1%)	
Yes	28 (27.2%)	1 (33.3%)	6 (23.1%)	3 (42.9%)	18 (26.8%)	
Parity						0.570
None	75 (72.8%)	2 (66.7%)	18 (69.2%)	4 (57.1%)	51 (76.1%)	
Once or more	28 (27.2%)	1 (33.3%)	8 (30.8%)	3 (42.9%)	16 (23.9%)	
PCOS	15 (14.6%)	0 (0%)	6 (23.1%)	2 (28.6%)	7 (10.4%)	0.219
Diagnosis						0.325
AEH	66 (64.1%)	2 (66.7%)	19 (73.1%)	6 (85.7%)	39 (58.2%)	
EEC	37 (35.9%)	1 (33.3%)	7 (26.9%)	1 (14.3%)	28 (41.8%)	
Treatment regimen						0.837
MA/MPA	62 (60.2%)	3 (100%)	15 (57.7%)	5 (71.4%)	39 (58.2%)	
MA/MPA+LNG-IUS	38 (36.9%)	0 (0%)	10 (38.5%)	2 (28.6%)	26 (38.8%)	
Others	3 (2.9%)	0 (0%)	1 (3.8%)	0 (0%)	2 (3.0%)	
Family history of cancer	9 (8.7%)	1 (33.3%)	4 (15.4%)	0 (0%)	4 (6.0%)	0.123
CA125(IQR, U/mL)	14.38 (11.10–19.20)	28.84 (11.56–46.12)	11.24 (7.94–1 8.90)	13.23 (10.67–17.54)	14.38 (11.00–19.83)	0.799

Abbreviations: BMI: Body mass index; PCOS: Polycystic ovary syndrome; AEH: Atypical endometrial hyperplasia; EEC: Endometrioid endometrial cancer; MA: Megestrol acetate; MPA: Medroxyprogesterone acetate; LNG-IUS: Levonorgestrel intrauterine device; CA125: Cancer antigen 125; IQR: Interquartile range; MMR: Mismatch repair.

Molecular classification was performed using the ProMisE algorithm on 103 cases of evaluable endometrial tissue. The majority of cases (67/103, 65.0%) were classified as p53wt, followed by 26 cases (25.2%) of MMRd, 3 cases (2.9%) of *POLE*-ultramutated, and 7 cases (6.8%) of p53abn. Among the patients with MMRd, 12 had MSH2 deletion alone (Figure 1). Additionally, 7 subjects exhibited strong and diffuse p53 expression by IHC (Figure 1), and none of them lacked p53 proteins. Three *POLE* mutations were identified at exon sites 9, 13, and 14, respectively. Among the three patients, one had both *POLE* mutations and p53 mutations and was classified in the *POLE*-ultramutated cluster, based on previous studies [29, 30].

### Comparison of oncologic outcomes across the four molecular subtypes

During the initial treatment phase, a total of 94 individuals demonstrated CR. The median interval of CR for these individuals was 4.5 months (IQR, 3.0–7.0). After 6 months of treatment, all patients were evaluated for efficacy, and the results showed that 65 individuals (63.1%) achieved CR, 24 (23.3%) achieved PR, 9 (8.7%) had SD, and 5 (4.9%) experienced PD. Following 9 months of treatment, 78 individuals (75.7%) achieved CR, 14 (13.6%) achieved PR, 5 (4.9%) had SD, and 6 (5.8%) experienced PD. Among these patients, 26 (27.7%) experienced recurrence after achieving CR, with a median interval of 25.5 months (IQR, 13.5–60.5). Out of the 103 patients, 16 (15.5%) ultimately required standard treatment, including hysterectomy, due to treatment failure. The median time from the initial visit to the final surgical treatment for these patients was 62 months (IQR, 6.75–84.50).

**Figure 3.**
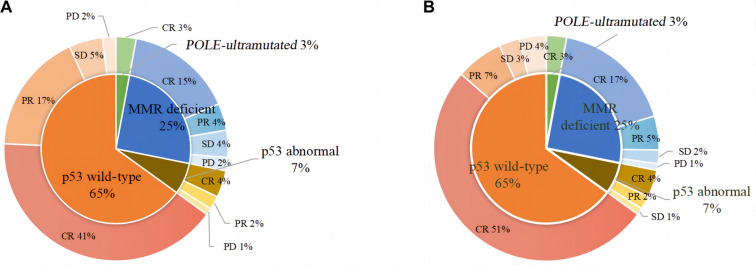
**Efficacy evaluation of patients with different molecular subtypes.** Oncologic outcomes of patients with different molecular subtypes at 6 (A) and 9 (B) months. Abbreviations: CR: Complete response; PR: Partial response; SD: Stable disease; PD: Progressive disease.

In patients with *POLE*-ultramutated, a CR rate of 100% was achieved after 6 months of regular efficacy assessment in fertility preservation therapy. The p53abn group had the lowest efficiency, with a CR rate of 57.1% after 9 months of regular assessment. The p53wt group showed improved efficacy compared to the MMRd group, with CR rates of 62.7% vs 61.5% and 79.1% vs 69.2% after 6 and 9 months of treatment, respectively (Table 2). However, there were no significant differences in the initial treatment outcome, treatment duration, CR rates at 6/9 months, and median time to CR (*P* > 0.05). The oncologic outcomes at 6/9 months are presented in Figure 3. Nonetheless, there were statistically significant differences in the rates of recurrence among patients with the four molecular subtypes (*P* ═ 0.010), with the lowest recurrence rate observed in the p53wt group at 19.7% and the highest recurrence rate in the p53abn group at 71.4%. The recurrence rates for *POLE*-ultramutated and MMRd were 66.7% and 30.4%, respectively. Additionally, there was a statistically significant difference (*P* ═ 0.001) in the need for standard surgical treatment due to fertility-preserving treatment failure, with the lowest rate of surgery observed in the p53wt group at 7.5%, compared to 66.7%, 19.2%, and 57.1% in the *POLE*-ultramutated group, MMRd group, and p53abn group, respectively. The treatment duration was categorized into two groups: ≤12 months and >12 months for stratified analysis (Tables S1 and S2). Significant differences in the proportion of patients with various molecular subtypes undergoing standard surgical treatment were observed in the subgroup with a fertility-preserving treatment duration of 12 months or less. The incidence rates were as follows: 66.7% for *POLE*-ultramutated, 17.6% for MMRd, 50.0% for p53abn, and 6.7% for p53wt (*P* ═ 0.007).

**Table 2:** Oncologic outcomes across molecular classification

**Variables**	**Total (*n* ═ 103)**	***POLE-*ultramutated** **(*n* ═ 3)**	**MMR deficient** **(*n* ═ 26)**	**p53 abnormal** **(*n* ═ 7)**	**p53 wild-type** **(*n* ═ 67)**	***P* value**
Overall CR rate (CR%)	94 (91.3%)	3 (100%)	23 (88.5%)	7 (100%)	61 (91%)	0.887
Oncologic outcome at 6 months						0.562
CR	65 (61.5%)	3 (100%)	16 (61.5%)	4 (57.1%)	42 (62.7%)	
PR	24 (23.3%)	0 (0%)	4 (15.4%)	2 (28.6%)	18 (26.9%)	
SD	9 (8.7%)	0 (0%)	4 (15.4%)	0 (0%)	5 (7.5%)	
PD	5 (4.9%)	0 (0%)	2 (7.7%)	1 (14.3%)	2 (3%)	
CR% at 6 months	65 (61.5%)	3 (100%)	16 (61.5%)	4 (57.1%)	42 (62.7%)	0.758
Oncologic outcome at 9 months						
CR	78 (75.7%)	3 (100%)	18 (69.2%)	4 (57.1%)	53 (79.1%)	
PR	14 (13.6%)	0 (0%)	5 (19.2%)	2 (28.6%)	7 (10.4%)	
SD	5 (4.9%)	0 (0%)	1 (20.0%)	1 (14.3%)	3 (4.5%)	
PD	6 (5.8%)	0 (0%)	2 (7.7%)	0 (0%)	4 (6.0%)	
CR% at 9 months	78 (75.7%)	3 (100%)	18 (69.2%)	4 (57.1%)	53 (79.1%)	0.381
Treatment duration (IQR, months)	12.0 (9.0–13.0)	9.0 (7.5–10.5)	12.0 (8.0–13.0)	12.0 (10.0–2 2.0)	11.0 (9.0–13.0)	0.456
Time interval to CR (IQR, months)	4.5 (3.0–7. 0)	3.0	4.0 (3.0–8. 0)	4.0 (3.0–20.0)	4.0 (3.0–7. 0)	0.732
Recurrence rate after CR	26 (27.7%)	2 (66.7%)	7 (30.40%)	5 (71.4%)	12 (19.7%)	0.010 ^*^
Time interval to recurrence (IQR, months)	25.5 (13.5–6 0.5)	29.0 (14.0–4 4.0)	21.0 (12.0–8 1.0)	54.0 (28.0–95.50)	19.0 (11.25–54.25)	0.537
Hysterectomy after treatment failure	16 (15.5%)	2 (66.7%)	5 (19.2%)	4 (57.1%)	5 (7.5%)	0.001^*^

*P < 0.05. Abbreviations: CR: Complete response; PR: Partial response; SD: Stable disease; PD: Progressive disease, IQR: Interquartile range; MMR: Mismatch repair.

**Figure 4.**
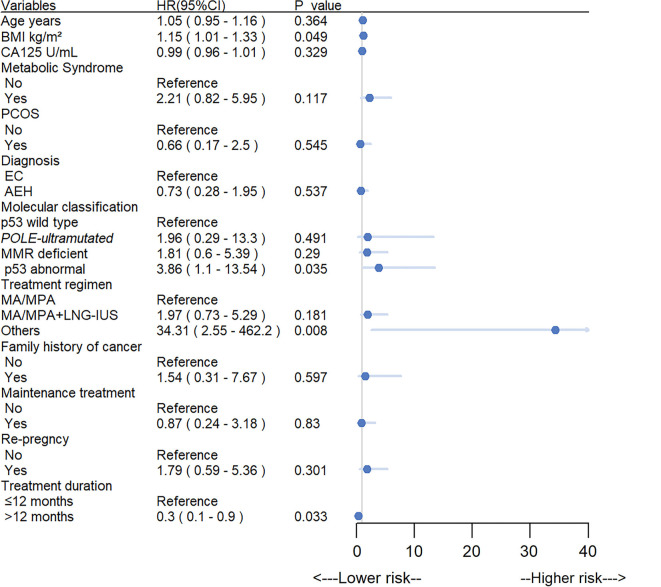
**Risk factors associated with recurrence.** Abbreviations: HR: Hazard ratio; CI: Confidence interval; BMI: Body mass index; PCOS: Polycystic ovary syndrome; AEH: Atypical endometrial hyperplasia; EEC: Endometrioid endometrial cancer; MA: Megestrol acetate; MPA: Medroxyprogesterone acetate; LNG-IUS: Levonorgestrel intrauterine device; CA125: Cancer antigen 125.

To investigate the relationship between clinicopathological indexes and recurrence, as well as to explore the risk factors, a comparison was made between the data of the recurrence group and the non-relapse group. Table S3 showed that molecular classification was significantly related to recurrence (*P* ═ 0.010). A multivariate analysis with Cox’s proportional hazard regression test was conducted, considering various factors such as age, BMI, molecular subtype, diagnosis, family history of cancer, treatment duration, maintenance therapy, re-pregnancy, initial treatment regimen, combination of MetS, and combination of PCOS as independent variables. The results of the multivariate Cox regression analysis indicated that BMI (HR 1.15, 95% CI 1.01–1.33, *P* ═ 0.049), p53abn subtype (HR 3.86, 95% CI 1.1–13.54, *P* ═ 0.035), and other treatment regimen (HR 34.31, 95% CI 2.55–462.2, *P* ═ 0.008) were associated with an increased risk of recurrence. On the other hand, a treatment duration of more than 12 months (HR 0.3, 95% CI 0.1–0.9, *P* ═ 0.033) was linked to a reduced risk of recurrence (Figure 4). Overall, patients with a p53abn tumor had the worst oncologic outcome.

### Comparison of fertility outcomes across the four molecular subtypes

The clinical information of the 49 patients who achieved complete remission (CR) and expressed a desire to conceive in the near future is presented in Table S4. Of these patients, 26 underwent ART, while the remaining 23 chose natural conception. As a result, 21 patients (42.9%) successfully became pregnant, which resulted in 11 (52.4%) live births, 5 (23.8%) miscarriages, and 5 (23.8%) ongoing pregnancies (Table 3). There were no significant differences in the choice of gestational options for patients with different molecular types (*P* ═ 0.957). There was a significant difference in pregnancy rates (*P* ═ 0.047) among patients with the four molecular subtypes. The pregnancy rate was highest in patients with p53wt (57.7%), followed by *POLE*-ultramutated (50%) and MMRd (31.5%). None of the patients with p53abn had a pregnancy. At the time of the last follow-up, 3 MMRd patients and 8 p53wt patients successfully delivered. One patient with MMRd and 4 patients with p53wt remained pregnant. Unfortunately, a *POLE*-ultramutated patient experienced an inevitable miscarriage of twins during their 22nd week of pregnancy.

**Table 3:** Fertility outcomes across molecular classification

**Variables**	**Total** **(*n* ═ 103)**	***POLE-*ultramutated (*n* ═ 3)**	**MMR deficient**	**p53 abnormal (*n* ═ 7)**	**p53 wild-type (*n* ═ 67)**	***P* value**
Desire to conceive after CR	49 (52.1%)	2 (66.7%)	16 (69.6%)	5 (71.4%)	26 (42.6%)	0.080
Pregnancy preparation method						0.957
Assisted reproduction	26 (53.1%)	1 (50%)	9 (56.3%)	3 (60.0%)	13 (50%)	
Nature conceived	23 (44.9%)	1 (50%)	7 (43.8%)	2 (40.0%)	13 (50%)	
Pregnancy rate	21 (42.9%)	1 (50%)	5 (35.1%)	0 (0%)	15 (57.7%)	0.047 ^*^
Pregnancy outcome						0.747
Live birth	11 (52.4%)	0 (0%)	3 (60.0%)	-	8 (53.3%)	
During pregnancy	5 (23.8%)	0 (0%)	1 (20.0%)	-	4 (26.7%)	
Abortion	5 (23.8%)	1 (100%)	1 (20.0%)	-	3 (20.0%)	

*P < 0.05. Abbreviations: MMR: Mismatch repair; CR: Complete response.

### *POLE*-ultramutated subtype and p53abn subtype

Among the patients with *POLE*-ultramutated, 2 initially had a diagnosis of AEH, and 1 had EEC. After three months of initial treatment, all 3 patients achieved CR. Two patients underwent standard surgical treatment for recurrence. Case 1 had a postoperative pathological diagnosis of AEH, while Case 2 had a postoperative pathological diagnosis of EEC stage IA G1. Among the three patients, only Case 2 experienced a spontaneous twin pregnancy after achieving CR, which unfortunately resulted in a miscarriage at 22 weeks. Among the patients with p53abn, 6 were initially diagnosed with AEH, and 1 had EEC. After 3–20 months of initial treatment, all 7 patients achieved CR. However, 5 patients experienced relapse after achieving CR. Among these, Cases 4, 5, 7, and 9 underwent standard surgical treatment for recurrence. Cases 4 and 5 had a postoperative pathological diagnosis of EEC stage IA G1, while Case 7 had synchronous endometrial and ovarian cancer (SEOC) stage IC G3. Case 9 had a postoperative pathological diagnosis of EEC stage IA G2-G3. One patient (Case 6) chose conservation treatment again after recurrence and achieved CR after 7 months. This patient is currently maintaining treatment. None of the 7 patients with p53abn type have successfully achieved pregnancy so far.

## Discussion

The diagnosis and treatment of EC have transitioned into an era of molecular detection. Scholars are increasingly studying the impact of molecular classification on fertility-sparing treatments. This single-institution study is the first to analyze both oncologic prognosis and fertility outcomes in relation to fertility-sparing treatment across four molecular subtypes. We observed significant differences in recurrence, surgery, and pregnancy rates among patients with EC/AEH who underwent fertility-sparing treatment with the four molecular subtypes (*P* < 0.05). Of these subtypes, p53wt appears to be the most favorable for fertility-preserving treatments.

The efficacy of fertility-sparing therapy in young patients with EC and AEH has been extensively studied. In our study, we observed a CR rate of 91.3% and a recurrence rate of 27.7%, aligning with the findings of previous studies [8–10]. However, it is important to acknowledge the significant variability in prognosis among patients. Therefore, the current research focused on identifying biomarkers that can accurately predict treatment responses and pregnancy outcomes. Since its introduction, the clinical utility of molecular classification has been extensively demonstrated in various studies over the past decade [14, 15, 31]. Furthermore, it has been shown that molecular classification is highly consistent between diagnostic endometrial and whole-uterus specimens [15, 32, 33]. These studies provide strong evidence that molecular classification can offer earlier and more reliable prognostic information for patients undergoing fertility-preserving treatment [34].

In our study, 26 (25.2%) participants were identified as MMRd, 3 (2.9%) as *POLE*-ultramutated, 67 (65%) as p53wt, and 7 (6.8%) as p53abn. Our findings align with those reported by Britton et al. [35], who also utilized the ProMisE classifier in a large cohort of young women with EC (<50 years old). However, our cohort included a smaller number of patients with *POLE* ultramutations. This discrepancy in results may be attributed to several factors. Firstly, most patients in our center presented with AEH (64.1%), which is characterized by less severe lesions. Additionally, our study included relatively young patients (<40 years). Another factor could be the limitations of Sanger sequencing, which can only detect mutations with an abundance greater than 30%. In contrast, their study sequenced *POLE* mutations in the *POLE* nucleic acid exonuclease structural domain (exons 9–14), whereas we only sequenced common mutations in exons 9, 13, and 14. Finally, their study included Asians (56%) and Caucasians (32%), whereas all patients in our study were Chinese–Asian. These factors may have contributed to the slight differences between our results and those of previous studies.

Within the ProMisE molecular classification, the *POLE*-ultramutated subtype exhibits the most favorable prognosis, whereas the p53abn subtype is associated with the worst prognosis. Conversely, the p53wt and MMRd subtypes demonstrate a moderate prognosis [31, 36, 37]. However, limited studies have been conducted on the relationship between molecular typing and conservation therapy. For example, Chung et al. examined a cohort of 57 patients with G1 to G2 EC and found that the MMRd group exhibited a lower CR/PR than that of the p53wt group. Moreover, the MMRd group showed a poor response to progestin treatment [20]. Puechl et al. reported the worst prognosis in patients with the p53abn subtype, with 22 cases of EC and 36 cases of AEH. Among the p53abn, MMRd, *POLE*-ultramutated, and p53wt groups, 50%, 33.3%, 25%, and 13.6% of the patients, respectively, experienced disease progression or required surgery [21]. In a study conducted in China by Zhang et al., the prognosis of 59 patients with AEH/EEC was compared using TCGA molecular classification. The study found that patients with CNH (p53abn) and MSI-H (MMRd) subtypes had worse prognoses than patients with CNL (p53wt) and *POLE*-ultramutated subtypes [23]. Our study supports these findings, as we found that patients with *POLE* ultramutations had the most favorable outcomes at 6 and 9 months of efficacy assessment, whereas patients with p53abn mutations fared the worst. Additionally, patients with the p53wt subtype had better outcomes than those with the MMRd subtype after undergoing fertility preservation therapy. Patients with the p53wt subtype had the lowest rates of recurrence and the lowest percentage of standard surgical interventions resulting from treatment failure (*P* < 0.05).

It is crucial to consider reproductive outcomes as important metrics. Our study is the first to evaluate the relationship between molecular typing and pregnancy outcomes after fertility-sparing treatments. We observed a significant difference in pregnancy rates (*P* ═ 0.047) among patients with the four molecular subtypes. The p53wt group had the highest pregnancy rate (57.7%), followed by the *POLE*-ultramutated group (50%) and the MMRd group (31.5%); none of the patients in the p53abn group achieved pregnancy. When considering both oncologic and pregnancy outcomes, our findings revealed that patients with p53wt mutations had the lowest recurrence (19.7%) and surgery (7.5%) rates. Interestingly, this group also had the highest pregnancy rate (57.7%) and a significantly higher live birth rate (53.3%). These results suggest that p53wt may be the most favorable molecular subtype for fertility-preserving treatments. Conversely, patients with p53abn mutations had the lowest pregnancy rate (0%) and the highest recurrence rate (71.4%). These findings indicate that the p53abn subtype may not be suitable for hormonal therapy. Moreover, multivariate Cox regression analysis showed that the p53abn molecular subtype was a risk factor for recurrence after conservation therapy when compared to the p53wt molecular subtype (*P* ═ 0.035). In the multivariate regression analysis, higher BMI (*P* ═ 0.049) and other treatment regimens (*P* ═ 0.008) were also associated with an increased risk of recurrence. Our results are consistent with those of several previous studies, suggesting that an overweight/obese status adversely affects conservation therapy [38–40]. In our cohort, three patients were diagnosed with both endometriosis and adenomyosis and received various treatment regimens including MA/MPA, IUS, and GnRH-a. The presence of this comorbidity has been suggested to potentially impact treatment effectiveness and reproductive outcomes. Prolonged treatment (≥12 months) (*P* ═ 0.033) was associated with lower recurrence rates. Dagher et al. [41] concluded that some patients may require a treatment duration longer than the 12 months suggested by the NCCN guidelines to achieve CR. Future studies should investigate the efficacy and safety of progestin therapy beyond the 12-month period [31, 36, 37, 42]. However, research on preserving reproductive function in patients with p53abn mutations is limited. Therefore, patients with p53abn mutations seeking to preserve fertility should receive comprehensive information about associated risks and undergo tailored treatment and rigorous follow-up care.

The pregnancy outcomes in our cohort were not satisfactory, which may be attributed to extensive damage to the uterine cavity caused by multiple hysteroscopy-assisted endometrial biopsies. In a previous study, we found that the uterine exfoliated cell chromosomal aneuploidy detector (UterCAD) can be utilized as a non-invasive method for the early detection of EC. This discovery provides a novel treatment option for patients who require regular monitoring after fertility-sparing treatment, thereby reducing the need for invasive procedures and minimizing uterine damage [43].

In this study, we examined the effects of the less common *POLE*-ultramutated subtype on fertility preservation therapy. Contrary to our expectations, we did not observe the anticipated improvements in tumor and reproductive outcomes in patients with this subtype. *POLE* is a DNA polymerase involved in DNA synthesis and replication, playing a crucial role in correcting replication errors during DNA strand elongation [44]. Patients with the *POLE*-ultramutated subtype tended to be younger, presented with stage I G3 clinical stage, and generally had better prognoses. However, previous studies have primarily focused on surgical survival data and have provided limited information about hormonal therapy. In the study by Chung et al. [20], two patients with *POLE* ultramutations received standard surgical treatment after PD or recurrence. Puechl et al. [21] noted that the proportion of patients with *POLE* mutations undergoing definitive treatment was notably high, second only to those with p53abn mutations. In our cohort, three patients had mutations in exons 9, 13, and 14 of *POLE*. All patients achieved CR after the initial therapy, but two patients experienced relapse at 44 and 14 months, respectively. The patient who relapsed at 44 months opted for standard therapy, and final pathological examination of the entire uterine specimen showed AEH. Another patient who desired to preserve fertility after the initial relapse chose to undergo re-care and achieved CR again after 3 months of treatment. The patient underwent assisted reproduction and had a successful twin pregnancy. Unfortunately, a miscarriage occurred at 22 weeks for personal reasons. Four years later, the patient relapsed and ultimately underwent standard surgical therapy. In addition to a mutation in exon 9 of *POLE*, this patient also presented with p53 mutations, possibly contributing to the poor prognosis. However, it is important to note that this observation may be biased because of the rare occurrence of *POLE* mutations. Therefore, there is an ongoing debate on whether the criteria for conservation therapy should be relaxed for patients with the *POLE* ultramutations.

It is worth noting that this study is the largest single-center investigation to date that utilizes molecular typing to evaluate prognostic outcomes in patients with EC and AEH undergoing fertility preservation. This study is groundbreaking in its exploration of fertility outcomes using molecular typing. However, the study still has some limitations. This is a retrospective study, which has some limitations, such as small sample size, missing data, various biases, and differing treatment methods. All these factors might lead to the need for further multicenter randomized controlled trials with larger sample sizes to verify the results. All patients in our study were of Chinese descent; therefore, caution should be exercised when extrapolating these results to other races or groups. In addition, the ER/PR status is critical for the development, treatment, and oncologic and fertility outcomes of EC patients. We did not assess the ER/PR status in this study, which might overstate the prognostic value of the molecular subclasses. Based on these shortcomings, we will design a multicenter randomized controlled trial with a larger sample size to investigate the outcomes of different molecular classifications. We hope our future study will yield more conclusive results.

## Conclusion

In conclusion, our findings suggest that ProMisE molecular classification is a valuable tool for predicting outcomes in patients with EC/AEH who undergo fertility-sparing treatment. Patients with p53wt mutations demonstrated promising results in terms of both oncological and reproductive outcomes following progestin treatment. Future studies should prioritize prospective investigations with larger multicenter cohorts to yield more robust, evidence-based data. Such studies will be critical for assessing the impact and value of molecular classification on fertility-sparing treatment for EC.

## Supplemental data

Supplemental data are available at the following link: https://www.bjbms.org/ojs/index.php/bjbms/article/view/12445/3995.

## Data Availability

All data generated or analyzed in this study are included in this published article.
